# Six Years of *Acinetobacter* Species in Critical Care: Carbapenem Resistance and Non-Susceptibility, Clinical Outcomes, and Lessons for Stewardship

**DOI:** 10.3390/antibiotics15030267

**Published:** 2026-03-04

**Authors:** Mircea Stoian, Leonard Azamfirei, Andrei Claudiu Stângaciu, Stefan Manea, Danusia Onisor, Andrei Manea, Andrei Cora, Alina Danilesco, Adrian Man, Adina Stoian

**Affiliations:** 1 Department of Anesthesia and Intensive Care, “George Emil Palade” University of Medicine, Pharmacy, Science, and Technology of Târgu Mureș, Gheorghe Marinescu Street No. 38, 540142 Târgu Mureș, Romania; mircea.stoian@umfst.ro (M.S.); leonard.azamfirei@umfst.ro (L.A.); 2 Intensive Care Unit, Mures Clinical County Hospital, Street Gheorghe Marinescu No 1, 540103 Târgu Mureș, Romania; andreistingaciu@yahoo.com (A.C.S.); alinka942@yahoo.com (A.D.); 3 Faculty of Medicine, “George Emil Palade” University of Medicine, Pharmacy, Science, and Technology of Târgu Mureș, Gheorghe Marinescu Street No. 38, 540142 Târgu Mureș, Romania; 4 Gastroenterology Department, “George Emil Palade” University of Medicine, Pharmacy, Sciences and Technology of Târgu Mures, 540142 Târgu Mureș, Romania; danusia.onisor@umfst.ro; 5 Department of Radiology, “George Emil Palade” University of Medicine, Pharmacy, Science, and Technology of Târgu Mureș, Gheorghe Marinescu Street No. 38, 540142 Târgu Mureș, Romania; andrei.manea@umfst.ro; 6 Doctoral School of Medicine and Pharmacy, “George Emil Palade” University of Medicine, Pharmacy, Science, and Technology of Târgu Mureș, Gheorghe Marinescu Street No. 38, 540142 Târgu Mureș, Romania; 7 Department of Radiology, County Emergency Clinical Hospital of Târgu Mureș, 54136 Târgu Mureș, Romania; cora.andrei@yahoo.com; 8 Department of Microbiology, “George Emil Palade” University of Medicine, Pharmacy, Sciences and Technology of Târgu Mureș, 540139 Târgu Mureș, Romania; adrian.man@umfst.ro; 9 Department of Pathophysiology, “George Emil Palade” University of Medicine, Pharmacy, Science, and Technology of Târgu Mureș, Gheorghe Marinescu Street No. 38, 540142 Târgu Mureș, Romania

**Keywords:** ICU epidemiology, phenotypic resistance, empiric therapy adequacy, time to active therapy, antimicrobial stewardship, multidrug resistance

## Abstract

**Background**: *Acinetobacter* spp., particularly *A. baumannii*, is a major intensive care unit (ICU) pathogen frequently associated with carbapenem non-susceptibility and delayed initiation of receipt of therapy. **Methods**: We conducted a single-center retrospective ICU cohort study in Romania (January 2019–December 2024) of adults with clinical cultures positive for *Acinetobacter* spp. (first isolate per patient). Susceptibility was interpreted per EUCAST. We assessed species distribution, carbapenem non-susceptibility, receipt of at least one in vitro active empiric agent, time to active therapy (TTAT, from index culture collection), early inflammatory biomarkers [neutrophile-to-lymphocyte ratio (NLR) and C-reactive protein (CRP)], and 30-day mortality. Predictors of mortality were evaluated using multivariable logistic regression and receiver operating characteristic (ROC) analysis. **Results**: A total of 234 episodes were included; *A. baumannii* accounted for 87.6% (205/234). Carbapenem non-susceptibility among *Acinetobacter* spp. isolates was 89.3% (209/234). Empiric antibiotics were initiated within 24 h in 95.7% of patients (224/234), yet only 49.6% (116/234) received at least one empiric agent later confirmed to be active. TTAT was 6 days (IQR 4–7), and active therapy within 72 h occurred in 8.5% (20/234). Thirty-day mortality was 73.1% (171/234) and did not differ between carbapenem non-susceptible (EUCAST I + R) and carbapenem-susceptible (EUCAST S) *A. baumannii* episodes (73.2% vs. 72.0%, *p* = 1.00). In multivariable analysis, age was independently associated with mortality (OR 1.36 per 10-year increase, 95% CI 1.04–1.90), with acceptable model discrimination (area under the curve = 0.74). Early NLR and CRP did not differ between carbapenem non-susceptible and carbapenem-susceptible *A. baumannii* episodes. **Conclusions**: In this ICU cohort, *A. baumannii* was the predominant species, and carbapenem non-susceptibility was highly prevalent. Despite early empiric therapy, receipt of at least one in vitro active agent was often delayed, and early inflammatory biomarkers had limited discriminatory value. These findings support locally tailored empiric strategies informed by local epidemiology and reinforce the need for improved diagnostics and stewardship interventions in high-burden ICU settings.

## 1. Introduction

*Acinetobacter* spp., particularly *A. baumannii*, are opportunistic Gram-negative pathogens characterized by environmental persistence and a remarkable ability to acquire antimicrobial resistance determinants. Among more than 30 recognized species, *A. baumannii* is the most clinically relevant and is responsible for the majority of healthcare-associated infections (HAIs) in critically ill patients [1,2,3].

Patients in intensive care units (ICUs) are at high risk for HAIs due to the high prevalence of invasive procedures and devices, immunosuppression, comorbidity, frailty, and advanced age. Over the past decade, advances in infection prevention and control have reduced the incidence of device-related HAIs. However, the overall rate of ICU-acquired infections remains high, further exacerbated by the emergence of multidrug-resistant (MDR) pathogens [4,5]. In this context, the ongoing emergence of new pathogens complicates treatment and threatens patient outcomes. The COVID-19 pandemic further highlighted these vulnerabilities by disrupting infection prevention measures, increasing exposure risks for caregivers, and challenging the maintenance of quality of care [6,7].

Among ICU-acquired pathogens, *Acinetobacter* spp., particularly carbapenem-resistant *A. baumannii* (CRAB), are leading causes of ventilator-associated pneumonia and bloodstream infections, with reported high incidence in some high-prevalence regions and mortality rates exceeding 40% [8,9]. Their persistence in the ICU environment, combined with limited therapeutic options, makes CRAB one of the most challenging threats for infection control and patient outcomes.

Globally, *A. baumannii* has evolved over the past three decades from a relatively uncommon colonizer into one of the most challenging MDR pathogens [10,11,12]. It is now a leading cause of ventilator-associated pneumonia, bloodstream infections, urinary tract infections, and wound infections among critically ill patients [4]. Reflecting its clinical impact and limited therapeutic options, the World Health Organization continues to classify CRAB as a top-tier “critical priority” pathogen on its global priority pathogens list [13].

Recent epidemiological studies highlight the alarming global prevalence of CRAB. In China, Liu et al. (2024) reported that 80.9% of ICU isolates in 2021 were CRAB, with the emergence of a highly resistant ST164 clone harboring blaNDM-1 and blaOXA–23 and exhibiting elevated MIC50/MIC90 values compared with global clone 2 lineages [14]. In the United States, Lodise et al. (2025) reported an incidence of ~1.19 *A. baumannii* cases per 100 hospital admissions, including 0.44 CRAB cases, with significantly higher mortality and length of stay among CRAB-infected patients [9]. Systematic reviews confirm that CRAB infections are associated with mortality rates of 40–60%, particularly in bacteremia and pneumonia [15,16,17].

In Europe, surveillance data from the European Antimicrobial Resistance Surveillance Network consistently report high rates of carbapenem resistance among *Acinetobacter* spp., with Southern and Eastern countries most affected [18]. Multicenter studies from Italy and Greece confirm mortality rates exceeding 50% among patients with CRAB bloodstream infections [19,20]. In Romania, a recent five-year analysis from Iasi documented that *Acinetobacter* spp. HAIs occur in up to 8% of cases annually, with over 80% occurring in ICUs [21]. However, national data remain fragmented, and few studies offer longitudinal insights into resistance dynamics.

The COVID-19 pandemic further amplified the burden of *Acinetobacter* spp. infections in ICUs, likely due to prolonged mechanical ventilation, extensive antibiotic exposure, and strained infection control practices [6,22,23]. Outbreaks reported during this period highlighted the vulnerability of ICUs and the limited availability of therapeutic options [24,25,26]. Beyond patient outcomes, CRAB infections substantially increase healthcare costs through longer ICU stays, greater use of last-resort antibiotics, and higher resource utilization [25,27]. From a public health perspective, the persistence of CRAB strains threatens the effectiveness of antibiotic stewardship and underscores the need for robust surveillance and prevention strategies [28].

Despite its global and European relevance, comprehensive longitudinal data from Romania and Central and Eastern Europe are scarce. Addressing this gap is essential to understand local epidemiology across pre-pandemic, pandemic, and post-pandemic periods, to guide empiric therapy, and to strengthen infection control policies. In addition to resistance surveillance, prior antibiotic exposure, and the timing and adequacy of antimicrobial therapy, the selection and clinical impact of *Acinetobacter* spp. are central to ICU infections. Early inflammatory markers derived from routine blood tests, such as the neutrophil-to-lymphocyte ratio (NLR), may provide pragmatic prognostic information and help contextualize outcomes when standardized severity scores are not consistently available.

This single-center retrospective cohort study analyzes six years (2019–2024) of *Acinetobacter* spp. isolates from a Romanian tertiary care hospital. The objectives were to characterize epidemiological trends and antimicrobial resistance profiles, with an emphasis on CRAB; to evaluate clinical outcomes, including all-cause mortality and length of stay; and to assess the adequacy of empiric antibiotic therapy. By integrating microbiological, therapeutic, and clinical perspectives, this study aims to provide evidence that may inform antimicrobial stewardship and infection control strategies in Central and Eastern Europe.

## 2. Results

### 2.1. Study Population and Baseline Characteristics

A total of 234 adult ICU patients with a first clinical isolate of *Acinetobacter* spp. were included in the study cohort from 2019 to 2024. Baseline demographic and clinical characteristics by epidemiological period are presented in Table 1. The median age was 68 years [IQR 59–76], and 152 patients (64.9%) were male. The median duration of mechanical ventilation was 12 days [IQR 7–20], and 219 patients (93.5%) received systemic antibiotic therapy before isolate identification. The median Carmeli score was 3 [IQR 2–3], and the median ICU length of stay was 15 days [IQR 9–23].

Clinical sample types are summarized in Table 1. Endotracheal aspirate was the most common specimen (161/234, 68.8%), followed by wound or other purulent secretions (41/234, 17.5%), blood cultures (24/234, 10.3%), and urine cultures (8/234, 3.4%). The 30-day mortality was assessed from the index culture date.

### 2.2. Species Distribution and Temporal Trend

**Figure 1:**
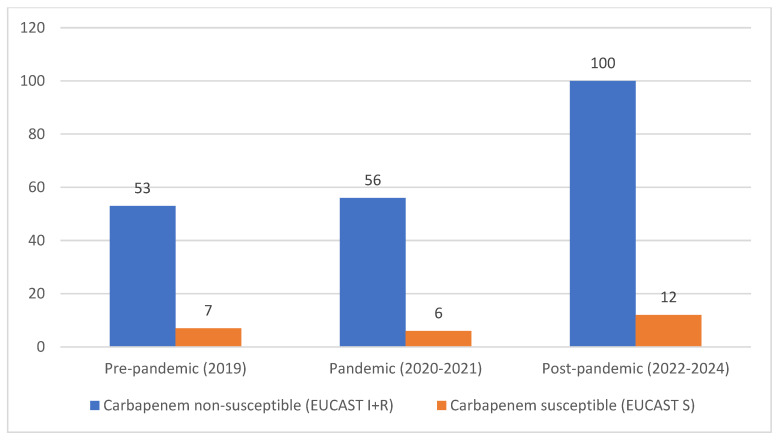
Temporal distribution of *Acinetobacter* spp. and CRAB across epidemiological periods. Note. Values represent the absolute numbers of isolates in each period. CRAB was assessed exclusively among *A. baumannii* isolates.

The annual distribution of *Acinetobacter* species and the carbapenem non-susceptibility profile among *Acinetobacter* spp. isolates [carbapenem non-susceptible *A. baumannii*) vs. (carbapenem-susceptible *A. baumannii*)] are shown in Table 2. Across the study period, *A. baumannii* accounted for the majority of isolates, whereas non-baumannii species accounted for a smaller proportion. The temporal distribution across epidemiological periods is illustrated in Figure 1, which presents the absolute annual episode counts of *A. baumannii*, non-baumannii *Acinetobacter* spp., and carbapenem-resistant *A. baumannii* (CRAB, EUCAST R). Incidence density rates per ICU admissions or per 1000 patient-days were not calculated due to the lack of consistently available denominator data across the study period.

### 2.3. Antimicrobial Susceptibility

Antimicrobial susceptibility testing results for *Acinetobacter* spp. isolates, interpreted using EUCAST criteria, are summarized in Table 3 as annual proportions categorized as EUCAST I (susceptible, increased exposure) or EUCAST R (resistant), reported together to reflect reduced susceptibility at standard exposure for carbapenems (imipenem and meropenem), fluoroquinolones (ciprofloxacin), aminoglycosides (gentamicin and amikacin), and colistin. Cephalosporins were not included in the susceptibility summary due to limited clinical relevance for *Acinetobacter* spp. and inconsistent routine testing/reporting in our setting. Cefiderocol susceptibility testing was not performed during the study period.

### 2.4. Empiric Antibiotic Therapy and Phenotypic Activity

Most patients received empiric systemic antibiotic therapy before isolate identification, with initiation within 24 h in 224 patients (95.7%). Overall, 116 patients (49.6%) received at least one empiric antimicrobial agent that was later confirmed to be in vitro active against the index isolate.

The median time to initiation of active therapy (TTAT), defined as the time from index culture collection to the first administration of at least one in vitro active agent, was 6 days [IQR 4–7]. Active therapy was initiated within 72 h in 20 patients (8.5%).

Adequacy and timing of empiric antibiotic therapy, stratified by carbapenem resistance category (EUCAST R vs. S/I), are presented in Table 4. TTAT was shorter in EUCAST R episodes compared to S/I episodes (median 5 vs. 6 days). However, the proportion of patients receiving at least one in vitro active agent within 72 h remained low in both resistance categories.

### 2.5. Clinical Outcomes and Predictors of 30-Day Mortality

In the overall *Acinetobacter* spp. cohort, 30-day all-cause mortality was 73.1% (171/234). Mortality did not differ between carbapenem non-susceptible (EUCAST I + R) and carbapenem-susceptible (EUCAST S) *A. baumannii* isolates (73.2% vs. 72.0%; *p* = 1.00).

As shown in Table 5, survivors had longer median hospital stay and longer duration of mechanical ventilation compared to non-survivors. Age was higher in the deceased group, whereas APACHE II, SOFA, and Carmeli scores did not differ significantly between groups. The proportion of carbapenem non-susceptible isolates was similar among survivors and non-survivors.

Logistic regression was used to identify independent predictors of 30-day mortality. Age was modeled as a continuous variable, scaled by 10-year increments, and sex was included as a covariate. Mechanical ventilation duration (days) was included as a clinical covariate; however, it was interpreted cautiously due to potential survivor (time-at-risk) bias. To reduce overfitting, a parsimonious model was constructed. Internal validation was performed using 1000 bootstrap resamples.

The multivariable model was statistically significant (χ^2^ = 43.28, *p* < 0.001) and explained 27.1% of the variance in 30-day mortality (Nagelkerke R^2^ = 0.271). Increasing age (per 10-year increment) was independently associated with higher mortality. Longer duration of mechanical ventilation was inversely associated with mortality; this inverse association likely reflects survivor (time-at-risk) bias, as patients who died early accumulated fewer ventilator days (Table 6).

**Figure 2:**
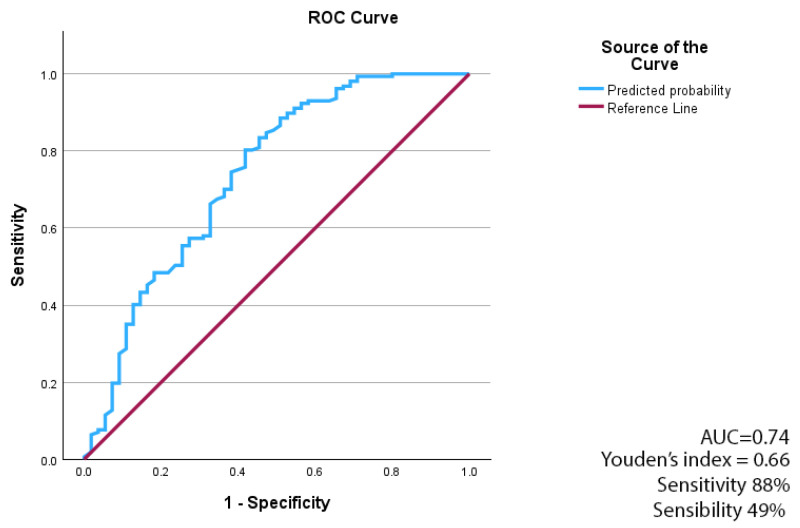
ROC curve for the multivariable logistic regression model predicting 30-day mortality. Note. The ROC curve was generated from predicted probabilities of the multivariable logistic regression model. Discrimination is quantified as AUC.

The discriminative ability of the multivariable logistic regression model was assessed using receiver operating characteristic (ROC) curve analysis. The model demonstrated acceptable discrimination, with an area under the ROC curve (AUC) of 0.74. Overall, the model demonstrated a moderate ability to distinguish between survivors and non-survivors at 30 days. The model achieved a sensitivity of approximately 88% and a specificity of approximately 49% for predicting 30-day mortality (Figure 2).

### 2.6. Early Inflammatory Markers and NLR Dynamics

Baseline and early inflammatory biomarkers were available at ICU admission, 24 h, and 72 h in a subset of patients. Median NLR and C-reactive protein (CRP) values at each time point are summarized in Table 7. No statistically significant differences in NLR or CRP were observed between CRAB and non-CRAB episodes at baseline, 24 h, or 72 h.

## 3. Discussion

### 3.1. Principal Findings

In this six-year retrospective ICU study, we characterized the epidemiology, antimicrobial susceptibility, and empiric antibiotic management of *Acinetobacter* spp. isolates, with a focus on carbapenem resistance and its clinical implications. *A. baumannii* was the predominant species, and carbapenem non-susceptibility remained common throughout the study period. Although empiric therapy was initiated early in most patients, fewer than half received at least one empiric agent later confirmed to be active, and time to active therapy was often prolonged, underscoring the difficulty of achieving timely, microbiologically adequate treatment in high-resistance settings.

Thirty-day mortality was high in the overall *Acinetobacter* spp. cohort and survivors had longer hospital stays and more ventilator days than non-survivors. Mortality did not differ between carbapenem non-susceptible and carbapenem-susceptible *A. baumannii* episodes. The absence of an observed association between carbapenem non-susceptibility and 30-day mortality should therefore be interpreted with caution. Given that a substantial proportion of isolates originated from respiratory specimens and that standardized infection criteria were not retrospectively applied, inclusion of colonized patients may have attenuated potential associations between resistance phenotypes and clinical outcomes. Although carbapenem resistance is clinically relevant, our findings suggest that early mortality in this ICU cohort was primarily driven by host-related factors and baseline severity, which may have outweighed the effect of resistance phenotypes. This absence of mortality difference should not be interpreted as biological equivalence between resistance phenotypes. Rather, it may reflect residual confounding by baseline illness severity and ICU case-mix, as mortality in critically ill populations is strongly influenced by host factors, clinical severity, and treatment limitation decisions rather than pathogen characteristics alone, as demonstrated in recent ICU outcome and end-of-life analyses [29,30]. Although ICU admission policies and decisions to forgo life-sustaining treatment were not systematically captured in our retrospective dataset, these contextual factors are recognized determinants of mortality patterns in critically ill populations.

In Table 1, diabetes mellitus and COPD differed significantly across epidemiological periods. These comorbidities are clinically relevant in critically ill populations and may contribute to vulnerability and unfavorable outcomes, although they were not evaluated as independent predictors in the adjusted mortality model. In addition, the distribution of sample sources differed across periods, with higher proportions of bloodstream and wound/other isolates in specific periods, which may reflect changes in case-mix and infection phenotype over time.

Early inflammatory biomarkers (NLR and CRP) did not differ between carbapenem non-susceptible (EUCAST I + R) and carbapenem-susceptible (EUCAST S) *A. baumannii* episodes during the first 72 h, indicating limited discriminatory value for early identification of carbapenem non-susceptibility.

Together, these results underscore the complexity of managing *Acinetobacter* infections in critical care and support the need for improved diagnostics, local epidemiological awareness, and stewardship-driven early therapeutic decisions.

### 3.2. Epidemiology of Acinetobacter spp. and Carbapenem Resistance

The predominance of *Acinetobacter baumannii* among *Acinetobacter* isolates in our ICU cohort aligns with findings from contemporary critical care studies, in which *A. baumannii* is the primary clinically relevant species linked to severe infections and antimicrobial resistance. The recent literature indicates that *Acinetobacter baumannii* accounts for most clinically significant *Acinetobacter*-related infections in critical care settings, particularly among mechanically ventilated patients and those with prolonged ICU stays [8,31,32]. Carbapenem non-susceptibility *A. baumannii* is commonly associated in the literature with OXA-type carbapenemases, efflux mechanisms, and permeability alterations. However, the present study did not include molecular characterization of resistance mechanisms; therefore, the underlying genetic determinants in our cohort cannot be established. Accordingly, these findings should be interpreted as phenotypic surveillance data reflecting local susceptibility patterns rather than molecular epidemiological characterization or clonal dissemination analysis.

The rapid dissemination of resistance is further facilitated by mobile genetic elements and clonal spread in hospital settings [33,34].

The sustained presence of carbapenem resistance among *A. baumannii* isolates over the six-year study period underscores the endemic nature of CRAB in high-acuity settings. Similar persistence of CRAB has been reported in multicenter ICU studies, reflecting both clonal dissemination and selective pressure from broad-spectrum antimicrobial exposure [35,36]. Although fluctuations in the absolute number of CRAB cases were observed across epidemiological periods, our study was not designed to formally assess temporal trends, so these variations should be interpreted descriptively.

Importantly, the epidemiological profile observed in our cohort mirrors patterns described in ICUs with a high burden of MDR organisms, where CRAB remains a leading cause of difficult-to-treat infections. These data reinforce the importance of continuous local surveillance, as regional variability in CRAB prevalence and resistance patterns can substantially influence empiric antibiotic decision-making in critical care.

### 3.3. Antimicrobial Susceptibility Patterns and Therapeutic Constraints

The antimicrobial susceptibility profile observed in our cohort, as summarized in Table 3, highlights the practical therapeutic constraints posed by MDR in *Acinetobacter baumannii* infections. Beyond carbapenem resistance, susceptibility to several commonly used antimicrobial classes was inconsistent, further narrowing empiric treatment options in the ICU. Comparable MDR phenotypes have been widely reported in recent critical care studies, underscoring the limited flexibility of standard empiric regimens in high-burden environments [37,38,39].

In our cohort, the median TTAT from the index culture collection was 6 days, and only 8.5% of patients received an in vitro active agent within 72 h. This delay may reflect the limited therapeutic options available for carbapenem non-susceptible *Acinetobacter* in ICU settings and highlights the potential value of rapid diagnostics and stewardship guided escalation strategies [40,41].

In recent years, novel agents and targeted approaches have been developed for difficult-to-treat *A. baumannii* infections, including cefiderocol and sulbactam-based strategies (e.g., sulbactam–durlobactam), although access and local availability remain variable. In our center, cefiderocol susceptibility testing was unavailable during the study period, limiting our ability to assess its potential role in CRAB management. These constraints underscore the need for stewardship-driven, case-by-case use of last-resort options in high-risk ICU infections [32,42,43].

The coexistence of resistance across multiple antibiotic classes complicates early therapeutic decision-making and increases the likelihood of discordant empiric therapy. In this context, reliance on conventional empirical protocols without incorporating local susceptibility data may delay access to effective treatment, particularly for patients at high risk of infection with resistant organisms.

These findings underscore the importance of dynamic, ICU-specific antibiograms and close collaboration among intensivists, infectious disease specialists, and antimicrobial stewardship teams to adapt empiric strategies to evolving resistance patterns.

It should be emphasized that in vitro susceptibility does not necessarily equate to clinical adequacy in critically ill patients, particularly for agents such as colistin and aminoglycosides, where pharmacokinetic variability and toxicity may influence therapeutic effectiveness. Accordingly, antimicrobial adequacy in this study reflects phenotypic susceptibility patterns rather than a comprehensive evaluation of PK/PD-optimized or clinically individualized treatment strategies.

### 3.4. Empiric Antibiotic Therapy and Timing to Active Treatment

Despite early initiation of empiric antibiotic therapy in the vast majority of patients, achieving timely microbiologically active treatment was challenging in our cohort. As shown in the empiric therapy adequacy and timing data (Table 4), fewer than half of patients received at least one empiric agent that was later confirmed to be active against the causative isolate. Moreover, the time to initiation of active therapy remained prolonged, with only a small proportion of patients receiving effective antimicrobial coverage within the first 72 h.

These findings align with previous critical care studies that report substantial delays in appropriate antimicrobial therapy in settings with a high prevalence of MDR organisms [44,45]. In particular, infections caused by carbapenem-resistant isolates (EUCAST R) are frequently associated with discordant empiric regimens, reflecting both limited therapeutic options and the difficulty of accurately predicting resistance patterns at treatment initiation [46,47].

The prolonged time to active therapy observed in our study highlights the limitations of conventional culture-based diagnostics and standard empiric protocols in high-burden ICUs. Delays in microbiological confirmation and susceptibility results may lead to extended exposure to ineffective agents, underscoring the potential role of rapid diagnostic techniques and stewardship-guided empiric strategies to shorten time to effective treatment.

From a clinical perspective, these data underscore the importance of integrating local epidemiological knowledge, patient-level risk stratification, and early reassessment of empiric regimens to improve the likelihood of timely initiation of active therapy, particularly in patients at increased risk for carbapenem-resistant *Acinetobacter* infection.

### 3.5. Early Inflammatory Biomarkers: Limited Discriminatory Value

In our cohort, commonly used early inflammatory biomarkers, including the NLR and CRP, did not distinguish between carbapenem non-susceptible (EUCAST I + R) and carbapenem-susceptible (EUCAST S) *A. baumannii* episodes during the first 72 h after ICU admission (Table 7). This finding suggests that early systemic inflammatory responses are largely driven by the severity of critical illness rather than by specific resistance phenotypes.

Previous studies have shown that both NLR and CRP are sensitive but nonspecific markers of inflammation in critically ill patients. In the context of severe infections and organ dysfunction, inflammatory activation may be substantial regardless of the underlying pathogen’s resistance profile, thereby limiting the ability of these biomarkers to support early etiological or resistance-oriented decision-making [48,49,50,51].

The lack of discriminatory value observed in our study aligns with reports that inflammatory markers perform poorly in distinguishing MDR infections from susceptible ones during the acute phase. Although NLR and CRP may retain prognostic relevance in selected settings, their utility for guiding early empiric antimicrobial choices, particularly regarding carbapenem resistance in *Acinetobacter* infections, appears limited.

Taken together, these findings support the view that relying solely on nonspecific inflammatory biomarkers is unlikely to improve the early identification of carbapenem-non-susceptible *A. baumannii* episodes. Instead, empiric strategies should be optimized and informed by local epidemiology, clinical risk factors, and, where available, rapid microbiological diagnostics.

### 3.6. Clinical Implications and Antimicrobial Stewardship

The findings of this study have several relevant clinical and stewardship implications for managing *Acinetobacter* infections in the ICU. The combination of a high prevalence of carbapenem non-susceptibility (EUCAST I + R), limited susceptibility to alternative agents, and delayed initiation of active therapy underscores the need for empiric strategies that are better aligned with local epidemiology and patient-specific risk profiles [52].

Although mechanical ventilation duration was inversely associated with 30-day mortality in the multivariable model, this association is best interpreted as a time-at-risk (survivor) effect, because early deaths inherently accrue fewer ventilator days. This highlights the limitations of using time-dependent ICU variables as linear predictors in retrospective mortality models.

Our results suggest that early empiric antibiotic initiation alone is insufficient to ensure adequate antimicrobial coverage in settings with a substantial burden of MDR organisms. In particular, the low proportion of patients receiving active empiric therapy and the prolonged time to effective treatment underscore the importance of early reassessment of empiric regimens once microbiological data are available. This reinforces the role of antimicrobial stewardship programs in supporting timely de-escalation or escalation decisions based on evolving clinical and laboratory information [53].

In high-risk ICU populations, integrating local resistance patterns, prior antimicrobial exposure, and established risk stratification tools into empiric decision-making may increase the likelihood of achieving early active therapy [54,55]. Additionally, the limited discriminatory value of nonspecific inflammatory biomarkers in our cohort underscores the need to rely on microbiological data and epidemiological context rather than inflammatory profiles alone when making empiric choices oriented toward carbapenem resistance and potential need for escalation [56].

Given limited access to novel antimicrobials and advanced susceptibility testing in many hospitals, their use should remain stewardship-controlled and be prioritized for extensive Drug Resistance/Pan-Drug Resistance *Acinetobacter* infections with few therapeutic alternatives. This approach aims to preserve activity and reduce unnecessary selection pressure in endemic ICU settings [52,57].

### 3.7. Limitations and Future Directions

This study has several limitations that should be considered when interpreting the findings. Its retrospective design is associated with potential missing data and unmeasured confounding, which may have influenced microbiological and clinical variables and precluded causal inference.

In addition, time-to-event analyses using Cox models with time-varying covariates or competing-risk approaches were not feasible due to the retrospective data structure, limited precision in exposure timing, and the relatively small number of events for stable modeling. Consequently, residual survivor (time-at-risk) bias cannot be fully excluded.

The absence of molecular characterization of carbapenem resistance mechanisms limits mechanistic interpretation and precludes assessment of potential clonal dissemination within the ICU. Consequently, stewardship and empiric therapy considerations derived from this study are based on phenotypic susceptibility patterns rather than on confirmed resistance genotypes.

Standardized illness severity scores were not available for all patients and were not fully integrated into the final adjusted model, which may have resulted in residual severity-related confounding. The use of complete-case analysis may introduce bias if the mechanism underlying the missing data was not completely at random. In addition, only the first *Acinetobacter* isolate per patient was included, potentially underestimating recurrent episodes or within-patient variability in resistance patterns.

A limitation of this retrospective study is the inability to consistently distinguish true infection from colonization, particularly in respiratory specimens. The relatively small number of bloodstream infections precluded statistically robust sensitivity analyses restricted to invasive infections. This may have diluted associations between resistance phenotype and outcomes, as mortality in this ICU cohort was likely driven primarily by baseline severity and comorbidities.

Furthermore, antimicrobial adequacy was defined solely on the basis of phenotypic susceptibility testing. Detailed evaluation of antibiotic classes, combination therapy patterns, dosing strategies, and drug-related toxicity was not available in the dataset. This limits assessment of true therapeutic appropriateness in critically ill ICU patients and restricts stewardship implications to phenotypic resistance patterns.

Early inflammatory biomarkers were not available at all predefined time points for all patients, which may limit the robustness of analyses involving NLR and CRP. Moreover, antimicrobial adequacy was defined solely on the basis of in vitro susceptibility results, without detailed assessment of dosing, exposure optimization, or pharmacokinetic/pharmacodynamic considerations.

Finally, this was a single-center ICU study, which may limit the generalizability of the findings in settings with different epidemiological and antimicrobial resistance profiles. Susceptibility testing for newer agents, such as cefiderocol, was not performed, limiting the evaluation of emerging treatment options for carbapenem-resistant *Acinetobacter* infections in our setting [52].

## 4. Materials and Methods

### 4.1. Study Design and Settings

This single-center, retrospective cohort study was conducted at Mures Clinical County Hospital, a tertiary-care university hospital in Romania, over a six-year period (January 2019–December 2024). The hospital has 1186 beds, including 44 in the Intensive Care Unit, and provides medical services for critically ill patients in the region. The study was approved by the institution’s ethics committee (approval no. 5200/2025).

### 4.2. Study Population

Inclusion criteria were age ≥ 18 years; at least one clinical culture positive for *Acinetobacter* spp. from blood, urine, respiratory samples (endotracheal aspirate or bronchoalveolar lavage), or wound or purulent secretions; and available clinical and microbiological data required for the predefined analyses.

Exclusion criteria included screening or surveillance cultures. For patients with multiple positive cultures, only the first *Acinetobacter* spp. isolate per patient was included in the analysis.

Clinical differentiation between colonization and true infection was not systematically performed within the predefined study design. Standardized diagnostic criteria (e.g., CDC definitions for ventilator-associated pneumonia or other infection syndromes) were not retrospectively applied. Isolate inclusion was based on microbiological recovery from clinical specimens in ICU patients, irrespective of formal infection classification.

### 4.3. Microbiological Methods

Species identification and antimicrobial susceptibility testing were performed using the VITEK 2 Compact system (bioMerieux, Marcy l’Étoile, France). Antimicrobial susceptibility results were interpreted as susceptible, intermediate (susceptible, increased exposure), or resistant according to the EUCAST criteria valid for the corresponding year. Carbapenem non-susceptible *A. baumannii* was defined as EUCAST category I or R to imipenem and/or meropenem. CRAB was defined as *A. baumannii* isolates categorized as EUCAST R to imipenem and/or meropenem. The primary microbiological outcome was the detection of CRAB [29]. For analyses of empiric antibiotic adequacy (Table 4), isolates were additionally grouped as carbapenem-resistant (EUCAST R) versus non-resistant (EUCAST S/I) based on imipenem and/or meropenem.

Cephalosporins were not included in the susceptibility analysis due to the intrinsic resistance profile of *Acinetobacter baumannii* to most cephalosporins and their limited therapeutic relevance in this setting. Ampicillin–sulbactam susceptibility was not systematically assessed during the study period, as it was not included in the routine VITEK 2 automated susceptibility panel.

For patients with multiple positive cultures, only the first *Acinetobacter* spp. isolate per patient during the same ICU admission was included in the analysis to avoid duplicate observations. Subsequent isolates from the same patient were excluded, regardless of changes in species identification or susceptibility profile. If multiple specimens were positive on the same date, the first isolate, as recorded in the laboratory’s registration system, was selected.

Molecular characterization of carbapenem resistance mechanisms (e.g., detection of carbapenemase genes such as blaOXA-type or blaNDM) was not performed, as this study relied exclusively on routine clinical microbiology data. Isolates were not systematically stored for subsequent molecular or clonal/epidemiological analysis.

### 4.4. Antibiotic Exposure and Therapy Definitions

Antibiotic exposure prior to *Acinetobacter* spp. isolation was defined as any systemic antimicrobial therapy given during the ICU stay before culture positivity. Post-isolation therapy was defined as antimicrobial treatment initiated or modified after microbiological identification and susceptibility results were available. Adequate therapy was defined as the administration of at least one antibiotic to which the isolate subsequently became susceptible, as determined by antimicrobial susceptibility testing.

Active therapy was defined exclusively on the basis of in vitro susceptibility according to EUCAST criteria. The retrospective dataset did not allow systematic evaluation of dosing optimization, pharmacokinetic/pharmacodynamic considerations, combination versus monotherapy strategies, sulbactam-containing regimens, or drug-related adverse effects.

### 4.5. Inflammatory Biomarkers and NLR

Routine inflammatory biomarkers were collected at three predefined time points: at ICU admission (baseline), 24 h, and 72 h. NLR was calculated as the absolute neutrophil count divided by the absolute lymphocyte count at each time point.

### 4.6. Statistical Analysis

Categorical variables were summarized as frequencies and percentages, and continuous variables were summarized as mean ± standard deviation if the distribution was Gaussian or as median and interquartile range (IQR) if the distribution was non-Gaussian. Gaussian distribution was assessed using the Shapiro–Wilk normality test. Comparisons were performed using the chi-square test or Fisher’s exact test for categorical variables. For continuous variables, Student’s *t*-test (for two independent variables) or ANOVA (for more than three independent variables) was used to compare central tendencies if the variables were normally distributed, or the Mann–Whitney U test (for two independent variables) or Kruskal–Wallis test (for three or more independent variables) if they were not normally distributed. Factors associated with 30-day mortality were evaluated using univariate and multivariate logistic regression. An ROC analysis was applied to the multivariate logistic regression model to determine the AUC and the optimal cutoff value using the Youden index. A *p*-value < 0.05 was considered statistically significant in all statistical tests. Analyses were conducted using IBM SPSS Statistics for Windows, version 31.0 (IBM Corp., Armonk, NY, USA).

Illness severity was assessed using available APACHE II and SOFA scores. These variables were examined descriptively and in exploratory univariate analyses. To minimize the risk of overfitting and model instability, and considering incomplete availability of severity scores in some records, the final multivariable model was constructed using a parsimonious approach based on clinically relevant covariates.

Missing data were handled using complete case analysis. No imputation procedures were performed. All statistical analyses were conducted using available data for each variable.

## 5. Conclusions

In this six-year retrospective ICU study, *Acinetobacter* spp., predominantly *A. baumannii*, were associated with a high burden of carbapenem non-susceptibility and high 30-day mortality. Despite early empiric therapy in most patients, receipt of at least one in vitro active agent was often delayed, underscoring persistent challenges in achieving timely, phenotypic coverage in high-resistance settings. In this cohort, early mortality likely reflects host-related factors and baseline severity rather than by resistance phenotype alone. Future prospective, multicenter studies incorporating rapid diagnostics and susceptibility testing for newer agents are needed to refine empiric strategies and support stewardship-guided early escalation or de-escalation decisions.

## Figures and Tables

**Table 1:** Baseline characteristics of the study cohort and distribution of sample types.

Variable	Overall (*n* = 234)	Pre-Pandemic 2019 (*n* = 60)	Pandemic 2020–2021 (*n* = 62)	Post-Pandemic 2022–2024 (*n* = 112)	*p*-Value
Age, years, mean ± SD	66.89 ± 12.87	66.8 ± 12.06	68.42 ± 10.96	66.09 ± 14.23	0.646
Male sex, *n* (%)	152 (64.9)	40 (66.6)	36 (58)	76 (67.8)	0.410
Mechanical ventilation, duration (days), median [IQR]	12 (7–20)	10 (6.75–20)	12 (7–17)	13.5 (6.25–22)	0.530
Antibiotic therapy prior to isolation, *n* (%)	219 (93.5)	57 (95)	60 (96.7)	102 (91)	0.297
Carmeli score [IQR]	3 (2–3)	3 (2–3)	3 (2–3)	3 (3–3)	0.154
Length of stay, median [IQR]	15 (9–23)	16.5 (10–26)	13 (10–18)	15 (8–25)	0.190
Cardiovascular disease, *n* (%)	150 (64.1)	31 (51.66)	42 (67.74)	77 (68.75)	0.066
Diabetes mellitus, *n* (%)	78 (33.3)	14 (23.33)	29 (46.77)	35 (31.25)	0.019
Chronic kidney disease, *n* (%)	26 (11.1)	3 (5)	8 (12.9)	15 (13.39)	0.216
COPD, *n* (%)	72 (30.8)	17 (28.33)	12 (19.35)	43 (38.39)	0.03
30-day all-cause mortality, *n* (%)	171 (73)	40 (66.66)	57 (91.93)	74 (66.07)	<0.001
Endotracheal aspirate, *n* (%)	161 (68.8)	44 (73.33)	45 (72.58)	72 (64.28)	0.390
Blood, *n* (%)	24 (10.3)	2 (3.33)	11 (17.74)	11 (9.82)	0.033
Urine, *n* (%)	8 (3.4)	0 (0)	3 (4.83)	5 (4.46)	0.234
Wound/Other, *n* (%)	41 (17.5)	14 (23.33)	3 (4.83)	24 (21.42)	0.004

**Table 2:** Annual distribution of *Acinetobacter* species and carbapenem non-susceptibility profile (carbapenem non-susceptible *A. baumannii* vs. carbapenem-susceptible *A. baumannii*) in ICU clinical isolates, 2019–2024.

Year	Total *Acinetobacter* spp.: (*n*)	*A. baumannii, n* (%)	*Non-baumannii, n* (%)	CNS *Acinetobacter* spp.: *n* (%)	CS *Acinetobacter* spp.: *n* (%)
2019	60	58 (96.7)	2 (3.3)	53 (86.3)	7 (11.7)
2020	10	5 (50.0)	5 (50.0)	10 (100)	0 (0)
2021	52	33 (63.5)	19 (36.5)	46 (88.5)	6 (11.5)
2022	50	48 (96.0)	2 (4)	44 (88)	6 (12)
2023	37	36 (97.3)	1 (2.7)	31 (83.8)	6 (16.2)
2024	25	25 (100.0)	0 (0.0)	25 (100)	0 (0)
Total	234	205 (87.6)	29 (12.4)	209 (89.3)	25 (10.7)

Note. *Acinetobacter* spp. included *A*. *baumannii* and *non-baumannii Acinetobacter* isolates. Carbapenem non-susceptibility was defined as EUCAST category I or R to imipenem and/or meropenem. Percentages are calculated per year based on the total number of *Acinetobacter* spp. isolates for that year. Abbreviations: ICU, intensive care unit; carbapenem non-susceptible *A. baumannii*, carbapenem-non-susceptible *Acinetobacter* spp.

**Table 3:** Antimicrobial non-susceptibility rates of *Acinetobacter* spp. isolates by year (EUCAST).

Year/ Period	Imipenem % (I + R)	Meropenem % (I + R)	Ciprofloxacin % (I + R)	Gentamicin % (I + R)	Amikacin % (I + R)	Colistin % (I + R)
2019	52 (86.6)	53 (88.3)	54 (90)	45 (75)	1 (1.6)	2 (3.3)
2020	10 (100)	10 (100)	10 (100)	8 (80)	9 (90)	0 (0)
2021	46 (88.4)	46 (88.4)	46 (88.4)	43 (82.6)	46 (88.4)	2 (3.8)
2022	44 (88)	44 (88)	45 (90)	40 (80)	29 (58)	3 (6)
2023	31 (83.7)	31 (83.7)	31 (83.7)	31 (83.7)	23 (62.1)	8 (21.6)
2024	24 (96)	24 (96)	24 (96)	24 (96)	21 (84)	4 (16)

Note. Values are shown as *n* (%) of isolates categorized as EUCAST I (susceptible, increased exposure) or R (resistant) among all *Acinetobacter* spp. isolates tested each year. Not all antibiotics were tested for all isolates each year. Cefiderocol susceptibility testing was not performed during the study period. Values represent the percentage of non-susceptible isolates (EUCAST intermediate plus resistant, I + R). Susceptible (S) percentages can be derived as the complement to 100% for each antimicrobial agent and period.

**Table 4:** Empiric antibiotic therapy and adequacy in *Acinetobacter* spp. episodes, stratified by carbapenem resistance (EUCAST R vs. S/I).

Variable	Overall (*n* = 234)	EUCAST S/I (*n* = 59)	EUCAST R (*n* = 175)	*p*-Value
Any empiric therapy ≤ 24 h, *n* (%)	224 (95.7)	58 (98.3)	166 (94.8)	0.458
At least one active agent at start, *n* (%)	116 (49.6)	30 (50.8)	86 (49.1)	0.881
TTAT, median [IQR], days	6 (4–7)	6 (5–8)	5 (4–7)	0.001
TTAT ≤ 72 h, *n* (%)	20 (8.5)	2 (3.3)	18 (10.2)	0.115

Note. Percentages are calculated within each resistance category among patients who received empiric therapy. Abbreviations: S/I, EUCAST susceptible (S) or susceptible, increased exposure (I) to imipenem and/or meropenem; R, EUCAST resistant to imipenem and/or meropenem; TTAT, time to active therapy; IQR, interquartile range.

**Table 5:** Clinical outcomes, baseline severity scores, and treatment characteristics in patients with *Acinetobacter* spp. episodes, stratified by 30-day survival status.

Variable	Alive at 30 Days (*n* = 63)	Deceased at 30 Days (*n* = 171)	*p*
Number of days in hospital	31 (11–44)	13 (9–19)	<0.001
Duration of mechanical ventilation (days)	21 (7–31)	11 (7–17)	<0.001
APACHE II score	21.5 ± 8.07	22.91 ± 6.89	0.321
SOFA score	8.10 ± 3.48	9.37 ± 3.36	0.394
CARMELI score	3 (3–3)	3 (2–3)	0.096
Days with empiric treatment	6 (4–7)	5 (4–7)	0.244
Total duration of antibiotic therapy (days)	10 (5–23)	4 (2–8)	<0.001
Age	62 (54–74)	70 (61–78)	0.028
Carbapenem non-susceptible *Acinetobacter* spp. (EUCAST I + R), *n* (%)	56 (88.88)	153 (89.47)	1.00

Note. Data are reported as the median (IQR) or mean ± SD, as appropriate. Comparisons were performed using the Mann–Whitney U test for continuous variables and χ^2^ or Fisher’s exact test for categorical variables. Variables such as hospital length of stay and duration of mechanical ventilation are time-dependent and may be affected by survivor (time-at-risk) bias.

**Table 6:** Independent predictors of 30-day mortality (multivariable logistic regression).

	B	OR (Exp(B))	95% CI for OR	*p*-Value
Sex	0.290	1.34	0.63–3.12	0.442
Age (per 10-year increase)	0.309	1.36	1.04–1.90	0.027
Duration of mechanical ventilation (days)	−0.087	0.92	0.88–0.94	<0.001

**Table 7:** Inflammatory biomarkers (NLR and CRP) at baseline, 24 h, and 72 h in CRAB versus non-CRAB episodes.

Biomarker	Carbapenem Non-Susceptible *A. baumannii* (EUCAST I + R)	Carbapenem-Susceptible *A. baumannii* (EUCAST S)	*p*-Value
NLR at baseline, median [IQR]	17.2 (8.2–33.0)	19.9 (10.6–36.3)	0.307
NLR at 24 h, median [IQR]	15.0 (7.43–28.7)	20.8 (7.04–38.6)	0.362
NLR at 72 h, median (IQR)	11.7 (6.5–26.7)	21.0 (5.2–50.5)	0.618
CRP at baseline (mg/L), median (IQR)	10.5 (5.4–15.7)	11.0 (5.2–17.8)	0.440
CRP at 24 h (mg/L), median (IQR)	11.1 (5.2–17.3)	11.7 (5.6–26.1)	0.536
CRP at 72 h (mg/L), median (IQR)	11.6 (4.7–17.3)	5.5 (2.8–17.2)	0.238

Note. NLR and CRP were calculated for the patients with available laboratory data. Carbapenem non-susceptible *A. baumannii* (EUCAST I + R); carbapenem-susceptible *A. baumannii* (EUCAST S) NLR—neutrophil-to-lymphocyte ratio; CRP—C-reactive protein; IQR—interquartile range; CRAB—carbapenem-resistant *Acinetobacter baumannii*; non-CRAB—carbapenem-susceptible *Acinetobacter baumannii*.

## Data Availability

The data will be shared upon request. The authors used Grammarly for grammar and spelling corrections.

## Abbreviations

The following abbreviations are used in this manuscript:

ICU	Intensive Care Unit
HAI	Healthcare-associated infection
CRAB	Carbapenem-resistant *Acinetobacter baumannii* (EUCAST R)
MDR	Multidrug-resistant
EUCAST	European Committee on Antimicrobial Susceptibility Testing
BAL	Bronchoalveolar lavage
NLR	Neutrophil-to-lymphocyte ratio
CRP	C-reactive protein
TTAT	Time to active therapy
IQR	Interquartile range
OR	Odds ratio
CI	Confidence interval
COPD	Chronic obstructive pulmonary disease
ROC	Receiver operating characteristic
AUC	Area under the curve
